# An Enhanced Understanding of the Powder Bed Fusion–Laser Beam Processing of Mg-Y_3.9wt%_-Nd_3wt%_-Zr_0.5wt%_ (WE43) Alloy through Thermodynamic Modeling and Experimental Characterization

**DOI:** 10.3390/ma15020417

**Published:** 2022-01-06

**Authors:** Hanna Nilsson Åhman, Lena Thorsson, Pelle Mellin, Greta Lindwall, Cecilia Persson

**Affiliations:** 1 Swerim AB, Isafjordsgatan 28A, 16440 Stockholm, Sweden; pelle.mellin@swerim.se; 2 Division of Biomedical Engineering, Department of Materials Science and Engineering, Uppsala University, Lägerhyddsvägen 1, 75120 Uppsala, Sweden; cecilia.persson@angstrom.uu.se; 3 Exmet AB, Isafjordsgatan 28A, 16440 Stockholm, Sweden; lena.thorsson@exmet.se; 4 Department of Materials Science and Engineering, KTH Royal Institute of Technology, Brinellvägen 23, 10044 Stockholm, Sweden; gretal@kth.se

**Keywords:** Powder Bed Fusion—Laser Beam (PBF–LB), magnesium alloys, WE43, biodegradable metals

## Abstract

Powder Bed Fusion–Laser Beam (PBF–LB) processing of magnesium (Mg) alloys is gaining increasing attention due to the possibility of producing complex biodegradable implants for improved healing of large bone defects. However, the understanding of the correlation between the PBF–LB process parameters and the microstructure formed in Mg alloys remains limited. Thus, the purpose of this study was to enhance the understanding of the effect of the PBF–LB process parameters on the microstructure of Mg alloys by investigating the applicability of computational thermodynamic modelling and verifying the results experimentally. Thus, PBF–LB process parameters were optimized for a Mg WE43 alloy (Mg-Y_3.__9wt%_-Nd_3wt%_-Zr_0.5wt%_) on a commercially available machine. Two sets of process parameters successfully produced sample densities >99.4%. Thermodynamic computations based on the Calphad method were employed to predict the phases present in the processed material. Phases experimentally established for both processing parameters included α-Mg, Y_2_O_3_, Mg_3_Nd, Mg_24_Y_5_ and hcp-Zr. Phases α-Mg, Mg_24_Y_5_ and hcp-Zr were also predicted by the calculations. In conclusion, the extent of the applicability of thermodynamic modeling was shown, and the understanding of the correlation between the PBF–LB process parameters and the formed microstructure was enhanced, thus increasing the viability of the PBF–LB process for Mg alloys.

## 1. Introduction

Magnesium (Mg) alloys are among the lightest structural metals on the market today, demonstrating good specific mechanical properties and excellent biocompatibility [1]. Furthermore, they are biodegradable and have mechanical properties close to those of bone, making them especially suitable for load-bearing bioresorbable orthopedic implants [2,3,4]. Today, autografts are the preferable option for treating large bone defects; however, their limited availability and associated donor site morbidity creates a need for artificial materials as a possible substitution [5,6]. Bioresorbable materials are a desirable option, as eliminating a second surgery used to removing a permanent implant lowers the risk of infections as well as the long-term risk of a peri-implant infection, and thus reduces the need for antibiotics, which is a pertinent problem in health care all over the world [4,7,8]. Patient discomfort and medical costs are of course reduced as well [9].

The Mg–RE (rare earth metals) family of alloys WE43 (Mg-Y_3.7–4.3wt%_-RE_2.4–4.4wt%_-Zr_0.4wt%_) has gained increasing attention for bioresorbable metal implants in the past two decades due to the possibility of achieving slower degradation rates with WE43 compared to other Mg alloys [3]. Mg alloys generally have poor corrosion resistance due to the low electrochemical stability of the Mg matrix, the strong cathodic activity of the secondary phases present, refs. [10,11,12], and the limited stability of the MgO surface film in aqueous solutions [13,14,15]. The solid solution of yttrium (Y) decreases the electrochemical activity of the Mg matrix and stabilizes the surface through the formation of Y_2_O_3_ [16,17,18]. Furthermore, secondary phases formed in the WE43 alloys have lower cathodic activity compared to phases present in other alloy systems, and are thus not as detrimental to the corrosion resistance [19]. In 2013, a patented version of the WE43 alloy was the first biodegradable metal to be made commercially available as bioresorbable metal screws to treat hallux valgus [20]. Since then, a number of bioresorbable Mg implants based on the WE43 family of alloys has been developed. However, they are limited to smaller devices such as the orthopedic screws based on powder extruded WE43 [21]. Exploring new production routes, and especially Powder Bed Fusion–Laser Beam (PBF–LB) processing, could expand the possible applications of biodegradable Mg alloys to include patient-specific implants for improved healing of large bone defects.

PBF–LB is an advanced additive manufacturing (AM) technology offering unique possibilities in the manufacturing of complex structures, enabling design optimization for maximized functionality [22,23,24]. Regarding orthopedic implants, AM is particularly interesting due to the possibility of producing patient-specific designs with optimized structures for cell proliferation and bone regeneration [25,26]. The PBF–LB process is already established as a viable option for the production of various types of orthopedic implants of metal alloys such as Ti-6Al-4V [22,27], with one of the best-known examples being Ti-6Al-4V hip implants with an optimized surface for improved secondary fixation through enhanced bone ingrowth [28,29]. However, to the best of the authors’ knowledge, there are no biodegradable metal implants commercially available for clinical use being produced via PBF–LB. PBF–LB of an Mg alloy WE43 would enable the production of biodegradable complex implants optimized for bone ingrowth, for the improved healing of large bone defects.

PBF–LB of Mg was first proven feasible by Ng et al. in 2010, who investigated the influence of the laser power and laser speed on the melting morphology of two Mg powders with varying particle size distribution using single laser tracks [30] Since then, the processing of various Mg alloys by PBF–LB has been reported [31,32,33,34]. The first study on PBF–LB processing of Mg alloy WE43 was published in 2015, where parts with a 90% density were produced on a custom built PBF–LB system [35] Bär et al. investigated the microstructure in and around the last melt pool of a WE43 alloy processed by PBF–LB [36] They observed an average grain size of 4.7 ± 0.4 µm and a weaker texture in grains formed in the last melt pool, while a stronger texture and grain size of 20.4 ± 0.4 µm was observed in the bulk of the material. The formation of equiaxed grains were related to the decreasing cooling rates towards the center of the melt pool, which allowed for homogenous nucleation to take place in this region. The formation of the highly textured larger grains was ascribed to directional grain growth taking place due to the heat treatment that occurs as a result of the laser scanning of subsequent layers. The mechanical properties were evaluated by Zumdick et al., who concluded that the printed WE43 had a similar tensile strength to the extruded material and was superior to cast WE43 [37]. Moreover, Li et al. [38] showed that WE43 scaffolds processed by PBF–LB experienced a 35% loss in yield strength, obtaining a yield strength of 13 MPa after 28 days of in vitro immersion in simulated body fluid. They also concluded that the WE43 scaffolds did not exhibit any in vitro cytotoxicity for human osteoblast-like cell line MG-63.

However, even though there are a number of studies showing promising results, the relationship between the processing parameters and the microstructure remains poorly understood. There is a large amount of variation in the reported processing parameters, as well as varying degrees of texture and average grain sizes varying from under 1 µm [37] to up to 35 µm [39]. Moreover, much of the research has been done on non-standardized or adapted printing equipment. In order to establish the PBF–LB process as a viable option for the production of biodegradable Mg implants of various sizes and shapes, a thorough understanding of the influence of the process on the microstructure is needed, especially for readily-available manufacturing systems.

Computational thermodynamics based on the Calphad (CALculations of Phase Diagrams) method have been adopted in the past in order to enhance the understanding of Mg alloys and to increase the possibilities to optimize processes and alloy design [40,41]. However, attempts to correlate the phases predicted using computational thermodynamics with the phases obtained in Mg alloys processed by AM have not previously been reported. Using the Scheil–Gulliver model, the phases formed under non-equilibrium solidification can be estimated, providing a first indication of the phases expected to be formed during the PBF–LB process [40]. The Scheil–Gulliver model has previously been applied in studies of solidification during welding [42] and PBF–LB processing of other metals [43,44].

In order to increase the understanding of the printing process of Mg alloy WE43, the aim of this study was therefore to investigate the influence of two different previously-optimized process parameters on the grain and grain size distribution, to assess the phases predicted using computational thermodynamics based on the Calphad method, and to compare it with the phases obtained experimentally. The resulting chemical composition and microstructures were correlated to the optimized processing parameters and compared with the virgin powder. The use of a commercially available machine (EOS M290) with no adaptations ensures the applicability of the results to future studies.

## 2. Materials and Methods

### 2.1. Gas-Atomized Powder of Mg Alloy WE43: Specification and Characterization

Gas-atomized powder of an Mg alloy, WE43, (NMD GmbH, Heemsen, Germany) with a particle size of 25–67 µm was processed by PBF–LB. The composition of the powder as supplied by the manufacturer is given in Table 1. The morphology of the powder was characterized by secondary electron images using a scanning electron microscope (SE-SEM) (Sigma 300 VP, Zeiss, Oberkochen, Germany) with an acceleration voltage of 5 kV and a current of 0.5 nA. The cross-section of the powder was investigated with light optical microscopy (LOM) (Leica DM IRM, Leica Microsystems GmbH, Wetzlar, Germany). To prepare the powder samples for cross-sectional investigation, the powder was cold mounted using EpoFix resin (Struers, Copenhagen, Denmark). The samples were ground in three steps with 1200 grit, 2500 grit and 4000 grit papers and subsequently polished using oxide polishing suspension (OPS) (MasterMet, Buehler, Lake Bluff, IL, USA) for 10 min. For chemical analysis of the powder, the cross section of the powder was gold-plated and investigated using backscatter electron imaging with SEM (BSE-SEM) energy dispersive spectroscopy (EDS) using an acceleration voltage of 10 kV and a beam current of 1.8 nA. The relative alloy content was measured on a map with an area measuring 1.1 mm × 0.8 mm for one hour. Phases present in the powder were characterized by X-ray diffraction (XRD) (Bruker D8 Discover, Bruker, Billerica, MA, USA).

### 2.2. Additive Manufacturing of Mg Alloy WE43

The WE43 powder was processed in a commercially available PBF-LB machine (EOS M290, EOS GmbH, Krailling, Germany) equipped with a Yb–fiber laser with a maximum power of 400 W, a laser focus diameter of 100 µm, and a maximum scan speed of 7.0 m/s. The machine design secures a laminar gas flow across the build area. An inert atmosphere of argon gas was applied, and the oxygen content was maintained below 0.1%. No adaptations of the PBF-LB system were made. The amount of energy applied in the material can be defined by the volumetric energy density (*ED*) [22], which is calculated using Equation (1)

(1) $$ED=\frac{P}{v*h*d}$$

where the variables include laser power (*P*), scanning speed (*v*), hatch distance (*h*), and powder layer thickness (*d*). The variables were adjusted in order to evaluate EDs ranging from 20–300 J/mm^3^, as this covers the processing window for most other alloys processed by PBF-LB [45]. The powder layer thickness was kept at 30 µm, while the hatch distance was varied from 30 µm to 100 µm, the power from 100 W to 300 W, and scanning speed 500–4500 mm/s. The scanning direction was rotated 67° between each layer to minimize the overlapping of scanning tracks between layers and thus create a more homogenized microstructure and residual stress distribution [46].

In order to establish the optimal process parameters, the samples were first visually inspected to establish exterior signs of overmelting or lack of fusion. The cross sections of the samples were investigated with LOM in order to identify possible internal defects. To prepare the specimens for LOM, the samples were ground in four steps (400 grit, 1200 grit, 2500 grit and 4000 grit paper) and subsequently polished for 10 min using OPS.

The two most successful process parameters with regards to external and internal effects were chosen for further evaluation. Five samples per process parameter setting measuring 30 × 10 × 10 mm were printed vertically.

### 2.3. Experimental Characterization of Additively-Manufactured Samples

The density of each group was measured by the buoyancy method based on Archimedes’ density principle using a Mettler Toledo density kit (Mettler Toledo, Columbus, OH, USA). To further study the general microstructure of the printed materials in LOM, the polished samples were etched with 2% Nital for 5 s. For further microstructural and chemical composition analysis, the samples were again ground with 4000 grit paper and polished with OPS for 20 min. The microstructure and the chemical composition were investigated using SEM, EDS and XRD. Finally, grain size and texture were analyzed by electron back scatter diffraction imaging (EBSD) (Nordlys HKL detector, Oxford Instruments, Abingdon, UK); the acceleration voltage was 10 kV and the beam current was set to 5 nA. A step size of 0.8 µm was used, and grain boundaries were defined as having a misorientation greater than 15°. The data were analyzed using Aztec Crystal 2.0 (Oxford Instruments, Abingdon, UK), with the average grain size being defined by the equivalent circular diameter, *d*. Equivalent circular diameter is a common approximated grain size descriptor defined as the diameter of an approximated circle having the same area (*A*) as the grain. Assuming that the grain is circular, *d* can be calculated using Equation (2):

(2) $$d=2\sqrt{\frac{A}{\pi }}$$

The average grain size is calculated here in two ways, using the number average (Equation (3)) and area weighted average (Equation (4)).

(3) $$\bar{d}=\frac{\sum_{i=0}^{n}d_{i}}{n}$$

(4) $${\bar{d}}_{Area}=\frac{1}{\sum_{i=0}^{n}A_{i}}\overset{n}{\underset{k=0}{\sum }}A_{i}d_{i}$$

The area weighted average is commonly implemented for EBSD data, as it is less sensitive to outliers and more closely related to the distribution of grain size [47,48]; both are included here for reference.

### 2.4. Computational Modelling of Mg Alloy WE43 Processed by PBF-LB

Computational thermodynamics based on the Calphad method was applied in order to predict the phases formed during the PBF-LB process. To describe the thermodynamic properties of a material, the Gibbs energy of its individual phases can be modeled with the help of Calphad as a function of composition, temperature, and pressure. Thermodynamic data that are experimentally established for binary, ternary and sometimes quaternary systems are applied to obtain the model parameters. By using extrapolation methods, the phase descriptions for the lower order system can be combined and used to predict the thermodynamic properties of multi-component, multi-phase systems.

The thermodynamic calculations in this work were performed for the alloying content obtained from the EDS map measurements from the different samples in the case of the printed samples, while for the powder the calculations were performed for the alloying content supplied by the producers.

The experimental results were compared with the predicted phase fraction using the Thermo-Calc software package [49] and the Thermo-Calc TCS Mg-based Alloys Database version 5.0 [50].

To predict how the WE43 alloy behaves upon solidification, the Scheil–Gulliver model as implemented in the Thermo-Calc software was applied. The Scheil–Gulliver model describes the redistribution of solutes during non-equilibrium solidification of multi-component metal systems. In the Scheil solidification simulations, it is assumed that no diffusion takes place in the solid phases, while the diffusion in the liquid phase is regarded as being infinitely fast.

### 2.5. Statistical Analysis

IBM SPSS Statistics (v 26, IBM Corp, Armonk, NY, USA) was used to perform statistical analyses. Any difference in density between samples produced by the EDs of 60 J/mm^3^ and 120 J/mm^3^ was assessed through a *t*-test for equal means. Because Levene’s test for equality of variances was significant, the test results where equal variances were not assumed were used. A statistically significant difference was considered to be *p* < 0.05.

## 3. Results

### 3.1. Powder Characteristics

**Figure 1:**
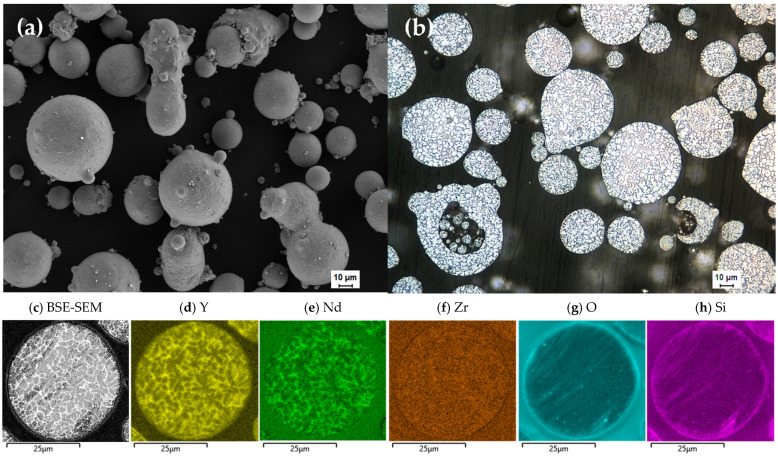
(**a**) SE-SEM image of the gas-atomized WE43 powder; (**b**) LOM image of the cross section of the powder; (**c**) BSE-SEM images of the cross section of the powder; and (**d**–**h**) EDS maps of Y, Nd, Zr, O and Si, respectively.

The SEM images of the gas-atomized WE43 Mg alloy powder (Figure 1a) showed spherical particles with some satellites and elongated particles present. Although the particles were not perfectly spherical, the powder exhibited good flow ability during the printing process, and the powder bed surface appeared smooth throughout. The LOM images of the cross-section of the powder (Figure 1b) showed some porosity, as well as the satellites seen in SE-SEM images. The BSE-SEM image of the cross section together with the EDS maps of the cross-section are presented in Figure 1c–g. A segregation of heavier elements can be observed in the BSE-SEM images. In the EDS maps, Nd and Y (Figure 1d,e, respectively) can both be observed in these regions, while the point analysis indicated that the brighter regions are mainly enriched in Nd. No regions rich in Zr were confirmed. An increase in O content around the surface as well as the bright spots appearing in the map is traces of the OPS used for polishing can be seen when comparing the EDS maps for O and Si. 

### 3.2. Experimental Characterization of Additively-Manufactured Samples

The WE43 powder was successfully processed by PBF-LB in the EOS M290 machine and the resulting microstructure was experimentally evaluated and compared with results from the computational thermodynamics. Some degree of evaporation was observed for all sets of process parameters. While the condensate products were to a large extent removed by the argon gas flow, some were deposited. The EDs below 50 J/mm^3^ resulted in non-dense samples due to lack of fusion, and above 150 J/mm^3^ the samples were overmelted or cracked. For the remaining EDs, samples with a limited number of defects were observed for a large variety of processing parameters. Two sets of process parameters with different EDs, presented in Table 2, were deemed successful with regard to the limited amount of external and internal defects, and were chosen for further evaluation. The densities for the two chosen process parameters are presented in Table 2. There was a statistically significant difference in density between the two groups (*p* = 0.029).

**Figure 2:**
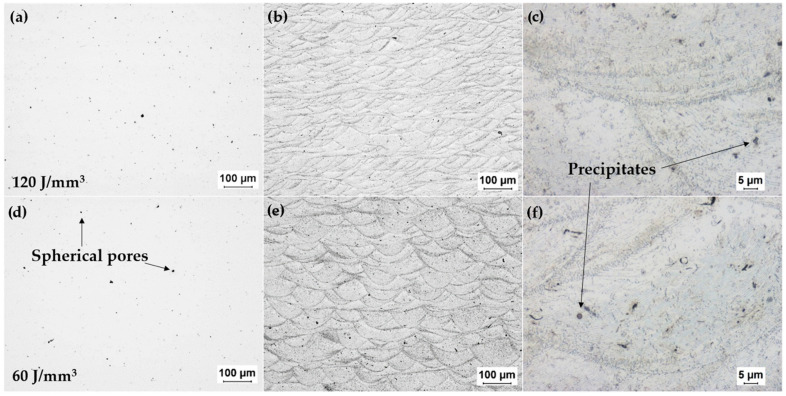
LOM images of the cross-sections parallel to the build direction of the two samples processed by PBF-LB (**a**) before and (**d**) after etching; (**b**,**c**,**e**,**f**), where (**a**–**c**) correspond to the samples processed with ED = 120 J/mm^3^, and the (**d**–**f**) correspond to the samples processed with a lower energy density of ED = 60 J/mm^3^.

The LOM images of the samples before and after etching can be seen in Figure 2b,c,e,f. Before etching, pores in the micrometer range as well as precipitates in the same size are visible. The pores present are of both spherical and irregular shape. Irregular pores are usually formed during the PBF-LB process due to lack of fusion, while circular pores can be related to the porosity of the powder, as seen in the LOM images, or to keyhole formation occurring due to an unstable melt pool during the printing process [51].

The LOM images of the samples after etching are presented in Figure 2b,e, in which the individual melt pools are clearly visible. The higher laser power of the 60 J/mm^3^ sample together with a larger hatch distance resulted in less overlap in the melt pools, thus penetrating deeper into the material [52]. Darker fields along the melt pool boundaries are visible for both energy densities, suggesting preferential etching and thus a higher concentration of precipitates in these regions. More overlapping of laser scanning tracks leads to more melt pool boundaries being present in the sample process with 120 J/mm^3^. A higher magnification of the LOM images (Figure 1c,f) of the two samples after etching highlights the similarities in the melt pool structures. In these two images, other defects such as inclusions and porosities are visible; however, it is difficult to distinguish them from each other in LOM images alone.

**Figure 3:**
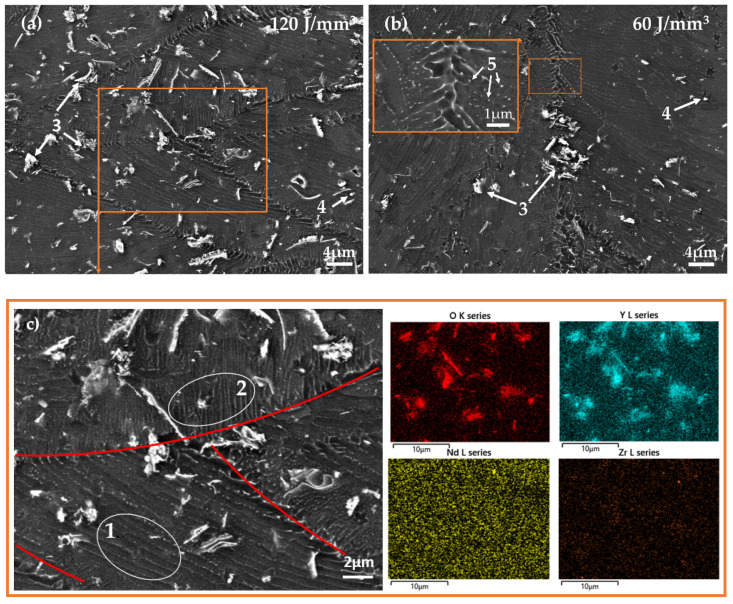
SEM images, where (**a**) corresponds to the samples processed with an energy density of 120 J/mm^3^ and (**b**) corresponds to the samples processed with an energy density of 60 J/mm^3^. The melt pool boundaries and the columnar structure are highlighted in image (**c**), corresponding to the boxed area in (**a**). The EDS maps correspond to the same area. Nr 1 and 2 highlights the orientation of the columnar structure observed in different parts of the melting pool. Nr 3 corresponds to areas rich in Y and O, and 4 to particles rich in Zr. Nr 5 are precipitates at the boundaries of the columnar structures.

The melt pools are also clearly visible for both samples in the SEM images. The melt pool boundaries are highlighted in red in Figure 3c, which displays a BSE-SEM image of the sample processed with 120 J/mm^3^. As was observed in the LOM images, the global structures are similar in both samples, with a varying amount of melt pool boundaries. In the image, lines of precipitates can be observed inside the melt pools parallel to the boundary of that melt pool (circled and marked *1* in Figure 3c). The distance between the lines varies from 500 nm to 1 µm, and is the same for both sets of processing parameters. Closer to the melt pool boundary at the bottom of the melt pool, columnar structures perpendicular to the boundary can be seen (circled and marked *2* in Figure 3c). The more random structure of the secondary phases found at the melt pool boundary is highlighted in the magnified insertion in Figure 3b. In Figure 3b, small precipitates marked *5* can be observed at the boundaries along the columnar cells. Furthermore, there are large flakes and brighter clusters appearing throughout the material, marked *3* in Figure 3a,b. A number of particles are present, appearing as white dots marked *4* in Figure 3a,b.

EDS mapping showed that the clusters and flakes were rich in O and Y in both sample groups [32,37]. Some speckles of high concentrations of Nd and Zr, respectively, could be discerned as well. EDS point analysis showed an increase in concentration of Nd and Y in the precipitates visible along the melt pool boundaries. These precipitates are identified in the literature as various Mg-RE intermetallics [37]. The point analysis showed a high Zr-content in the bright particles marked *4* in Figure 3. As Zr has a very low solubility in Mg and no tendency to form intermetallic compounds with the other alloying elements, these are likely to be pure Zr particles [2].

Comparing the general alloy content of the processed material obtained with EDS with the alloy content given for the powder (Table 3), there is an indication of a higher content of alloying elements in the processed material. This could be explained by an expected preferential evaporation of Mg related to the small difference between the melting and boiling points of Mg (T_melt_ (Mg) = 650 °C and T_boil_ (Mg) = 1050 °C) [53] compared with the high boiling point of the alloying elements (T_boil_ (Y) = 3338 °C, T_boil_ (Nd) = 3074 °C and T_boil_ (Zr) = 4377 °C) [53].

**Figure 4:**
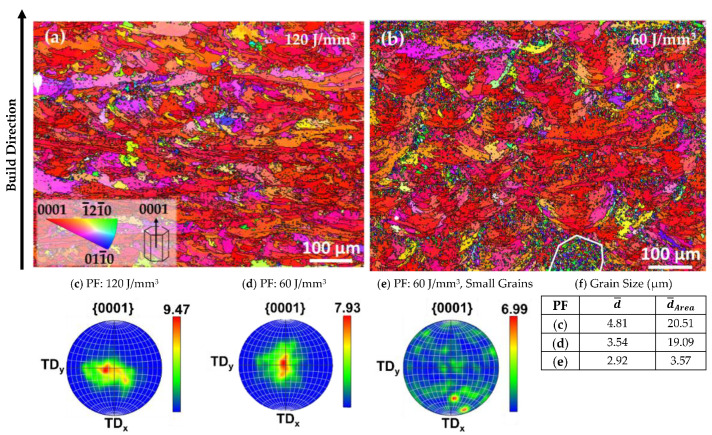
EBSD orientation map of (**a**) a sample produced with 120 J/mm^3^, (**b**) a sample produced with 60 J/mm^3^, with an area with a smaller grain size circled. An IPF color key valid for both maps is inserted in (**a**). In both maps, the IPF color is set to represent the crystallographic direction in the transverse plane in order to highlight the strong basal texture. This corresponds to the grains having the crystallographic orientation represented in the schematics of the hcp unit cell, i.e., a red colour means a crystal orientation of 0001 in the build direction. (**c**–**e**) include the PFs for the 120 J/mm^3^ sample, 60 J/mm^3^ sample, and the area with smaller grains circled in (**b**,**f**), respectively. The grain sizes are defined for the areas related to the three PFs. The build direction is upward in the images.

The EBSD images of the two sample groups are presented in Figure 4, together with the pole figures (PFs) corresponding to the two images and the PF corresponding to the area circled in Figure 4b. A strong basal texture is prevalent in the EBSD images for both samples, indicating a columnar grain growth where the grains have grown with the {0001} direction of the hexagonal closed packed (hcp) structure parallel to the build direction [54]. The {0001} direction of the hcp unit cell is indicated in the insert of Figure 4a. In the EBSD image of the 120 J/mm^3^ sample (Figure 3a), the individual melt pools are not as prevalent as in the LOM images in Figure 2, although a layered structure can clearly be discerned. Looking at the PFs in Figure 4c,d, the 120 J/mm^3^ sample exhibits a slightly stronger texture than the 60 J/mm^3^ sample. In the EBSD image for the 60 J/mm^3^ sample, Figure 4b, larger grains with shapes more clearly contained within the melt pool boundaries have been formed, with clusters of smaller equiaxed grains around them. A cluster of smaller grains is circled in Figure 4b, and the PF of the corresponding area shows that the small grains do not exhibit the same strong basal texture as the sample as a whole.

The numerical average $\bar{d}$ and the area weighted average ${\bar{d}}_{Area}$ for the grain size of the two samples, as well as the cluster of smaller grains circled in Figure 4b, are presented in the table in Figure 4f. The grains in the samples produced with 120 J/mm^3^ exhibit a larger average grain size than in the sample produced with 60 J/mm^3^. This can be related to the large amount of smaller equiaxed grains in the sample produced with 60 J/mm^3^.

**Figure 5:**
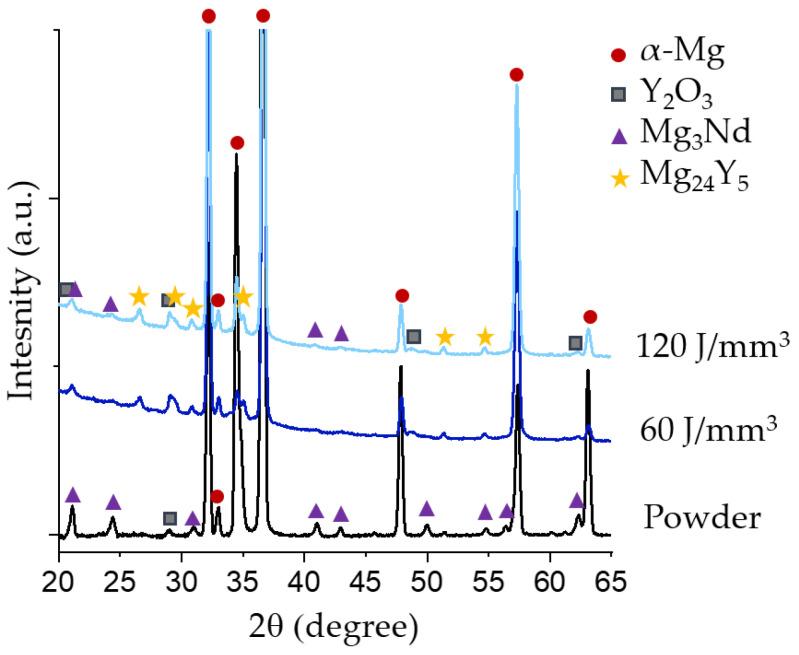
XRD spectra of the WE43 gas-atomized powder and the PBF-LB processed material.

No differences were observed between the XRD spectra obtained for the two samples (Figure 5). Analyzing the XRD spectra, most peaks correspond to α-Mg. Peaks corresponding to Y_2_O_3_ were found in the spectra for the processed material, although not in the spectra of the powder. The oxygen- and yttrium-rich regions observed in the EDS are thus likely be Y_2_O_3._ Peaks corresponding to Mg_3_Nd were found in both spectrums. Moreover, in the printed material a number of peaks and shoulders that could be related to Mg_24_Y_5_ were established. The difference in relative intensity between the α-Mg peaks in the spectra of the processed material is different from that of the powder. This change in relative intensity of the peaks is related to the strong texture in the processed material [55].

### 3.3. Computational Modelling Results

**Figure 6:**
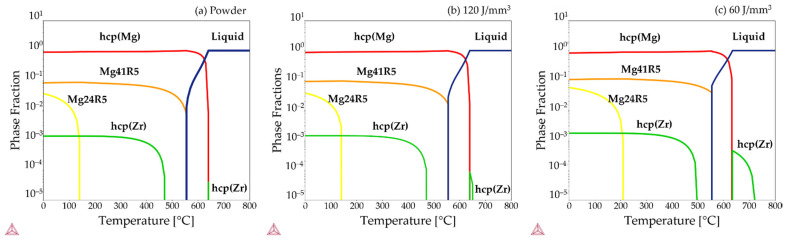
Calculated property diagrams showing the equilibrium phase fraction as a function of temperature for the alloying content given in Table 3 for (**a**) the powder, (**b**) the sample printed with ED = 120 J/mm^3^, and (**c**) the sample printed with ED = 60 J/mm^3^, using the Thermo-Calc TCS Mg-based alloys Database version 5.0 [49,50].

The calculated property diagrams with the equilibrium phase fractions as a function of temperature are shown in Figure 6a–c for the compositions quoted in Table 3 for the powder and for the material printed with 120 J/mm^3^, and 60 J/mm^3^, respectively. For all cases, the equilibrium phases are hcp-Mg and the Mg-RE rich intermetallic phases are Mg41R5 and Mg24R5 (with R = Nd and/or Y), together with low amounts of hcp-Zr. hcp-Mg is the α-Mg, Mg41R5 corresponds to Mg_41_Y_5_, and Mg24R5 corresponds to Mg_24_Nd_5_ [56,57]. For all compositions given in Table 3, a small fraction of hcp-Zr appears to be stable above the melting temperature of magnesium. This fraction increases with increasing amount of Zr. Apart from the higher melting point of hcp-Zr and a higher solvus temperature for of Mg_24_Nd_5_, the higher alloying content quoted for the 120 J/mm^3^ sample in comparison to the others in Table 3 does not have a major influence on the equilibrium phase fractions.

**Figure 7:**
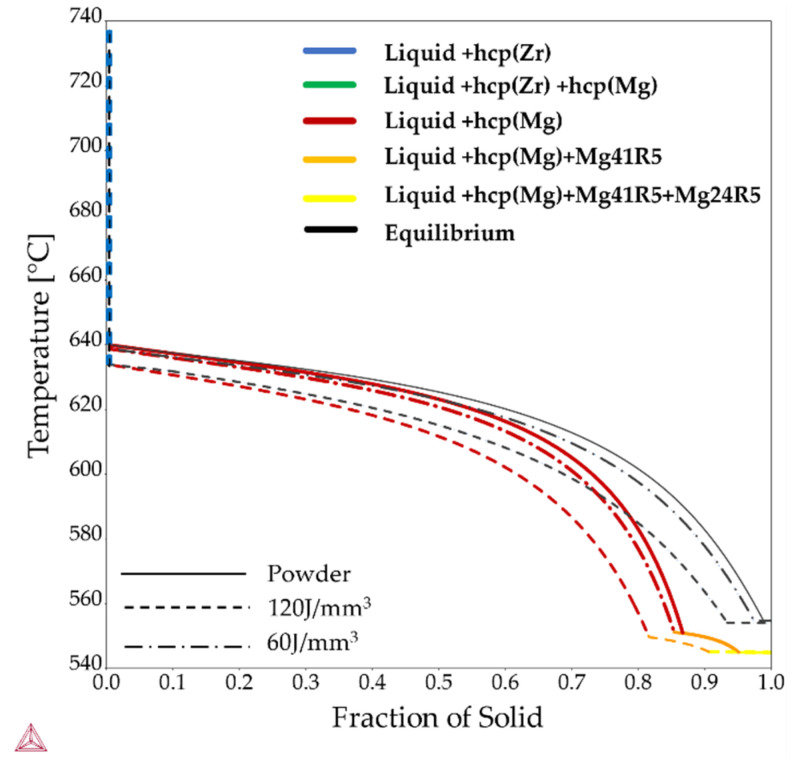
Scheil–Gulliver simulation showing the solidification paths in comparison to equilibrium for the alloying contents given in Table 3, using the Thermo-Calc TCS Mg-based alloys Database version 5.0 [49,50]. The higher thermal stability of the hcp-Zr observed in the equilibrium phase diagram is also visible in the calculated results of Scheil–Gulliver simulations for the sample printed with 120 J/mm^3^, where it corresponds to the green line. However, the temperature interval was too small to be visible in the graph.

To predict how the WE43 alloy behaves upon solidification under non-equilibrium conditions, the Scheil–Gulliver model as implemented in the Thermo-Calc software was applied. The compositions implemented in the model are the values presented in Table 3, and the results can be seen in Figure 7. The resulting solidification sequence for all three compositions is that the solidification of hcp-Zr takes place first, followed by the solidification of α-Mg. Finally, the solidification of Mg_41_Y_5_ takes place, followed by Mg_24_Nd_5_. The higher thermal stability of the hcp-Zr observed in the equilibrium phase diagram is visible in the calculated results of the Scheil–Gulliver simulations for the sample printed with 120 J/mm^3^, where it corresponds to the green line. However, the temperature interval was too small to be visible in Figure 7. The intervals where the hcp-Zr is present for the composition given for the powder and the sample printed with 60 J/mm^3^ are so small that they are hardly visible in the graph in Figure 7.

## 4. Discussion

Mg WE43 parts with above 99.4% density were successfully produced with PBF-LB from Mg-Y_3.9wt%_-Nd_3wt%_-Zr_0.5wt%_ powder processed with EDs of 120 J/mm^3^ and 60 J/mm^3^. The individual melt pools were clearly visible after etching and in the BSE-SEM images. The distribution of secondary phases was similar for both processing conditions and to the distribution observed in other studies [36,39,58]. The grain size and grain size distribution will be further discussed below. The phases predicted using thermodynamic modelling based on the Calphad technique included α-Mg, Mg_24_Y_5_, Mg_24_Nd_5_ and hcp-Zr. The phases experimentally established for both processing parameters included α-Mg as the main phase, along with Y_2_O_3_, Mg_3_Nd, Mg_24_Y_5_ and hcp-Zr. Hence, in comparing the computational and experimental results the phases α-Mg, Mg_24_Y_5_, and hcp-Zr were both predicted and experimentally observed. The phases Mg_24_Y_5_ and Mg_24_Nd_5_ were both predicted to be formed using the Scheil–Gulliver model. These phases have also been confirmed for both cast and PBF-LB processed WE43 in other studies [36,37,38,39,58]. A number of precipitates were shown to be high in both Y and Nd using EDS; however, only Mg_24_Y_5_ was confirmed through XRD. Mg_24_Nd_5_ is an established equilibrium phase in the Mg-Y-Nd ternary system. However, as the peaks for these two compounds overlap in the XRD spectra, the conclusions on which are present can vary between studies [36,37,38,39,58]. It is likely that both phases are present in the material.

**Figure 8:**
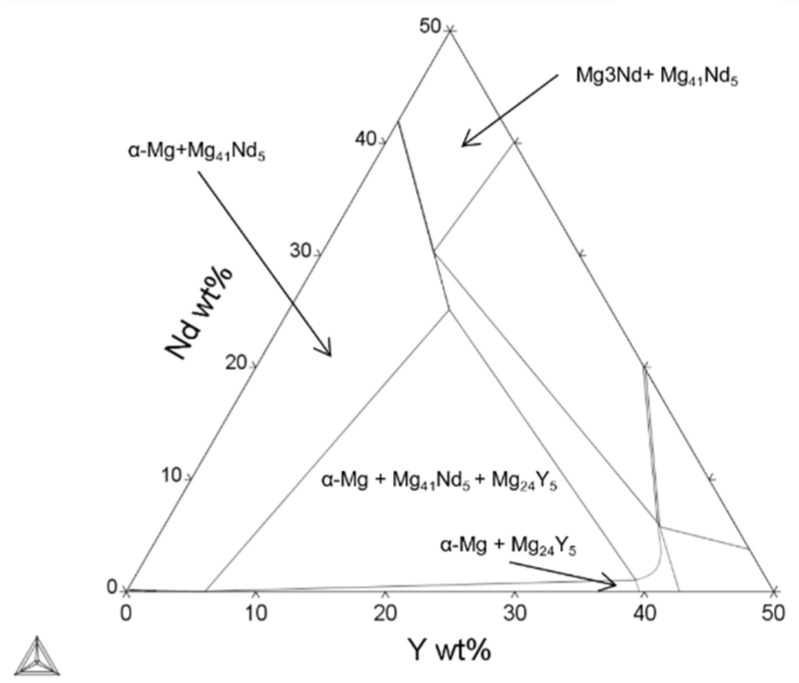
Calculated Mg-Y-Nd isothermal ternary phase diagram, T = 300 °C [49,50].

One phase that was not predicted using the Scheil–Gulliver model but was established using XRD was Mg_3_Nd. Mg_3_Nd is present as a thermodynamically stable phase at higher Nd concentrations in the ternary Mg-Y-Nd phases diagram (Figure 8). However, it has also been shown to be the first metastable phase to precipitate in low-alloyed Mg-Nd alloys (Nd = 2.53 wt%) [57], and the first phase to precipitate in ternary Mg-Y-Nd alloying systems [59]. This would also explain the large number of peaks corresponding to Mg_3_Nd in the spectra for the powder, as the fast cooling rates during the gas atomization would not allow enough time for the metastable phases to transform into equilibrium phases. Over time, the Mg_3_Nd is expected to transform first into Mg_12_Nd and finally the equilibrium phase, Mg_41_Nd_5_ [57]. Nie et al. [59] suggested the formation of Mg-Y-Nd ternary phases such as Mg_14_Nd_2_Y and Mg_12_NdY, which were observed in a PBF-LB processed WE43 by Bär et al. [36]. These phases were not obtained with neither computational nor experimental methods in the present study, and would be interesting to investigate in future studies. Moreover, the cooling rates present in the PBF-LB process are in the order of 10^6^ K/s [60]. At such high cooling rates, deviations from local equilibrium at the liquid–solid interface can be expected due to finite interface kinetics and solute trapping. These effects are not accounted for in the applied Scheil–Gulliver model, and hence certain metastable phases such as the Mg_3_Nd phase may not have been predicted by the current model.

**Figure 9:**
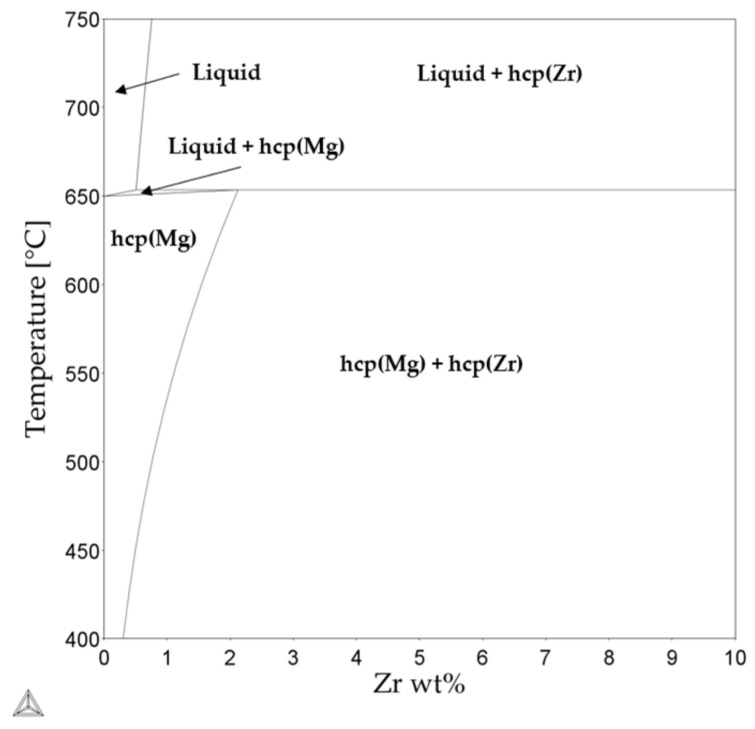
Calculated Mg-Zr binary phase diagram [49,50].

As there is no evidence of Zr rich forming intermetallic compounds in the Mg-Y-Nd-Zr system and due to the low solubility of Zr in α-Mg, the Zr particles observed with EDS are assumed to be hcp-Zr [54]. Zr is a common nucleation agent added to Mg-alloys to obtain grain refinement; thus, there are many studies investigating the precipitation of hcp-Zr particles and its influence over the solidification behavior of Zr containing Mg alloys [61,62,63]. The experimental investigations carried out on the binary Mg–Zr system shows the precipitation of the hcp-Zr in the liquid melt as well as the dissolution of hcp-Zr in the α-Mg matrix [64], which can be seen in the Mg-Zr binary phase diagram (Figure 9) as well. With respect to the amount of Zr in the alloy investigated here, hcp-Zr is expected, as predicted by the equilibrium and solidification calculations. However, the mechanisms by which precipitation and dissolution of the hcp-Zr particles occurs and the mechanism behind their effect as a grain refiner in Mg alloys is complex and not very well understood. Thus, more data are needed in order to accurately describe the precipitation behavior of Zr in this system.

The presence of Y_2_O_3_ was confirmed through EDS and XRD, and Y_2_O_3_ flakes have been confirmed by several studies on WE43 [36,39,52]. However, modelling the formation of oxides is highly complex, and was not included in the current work. MgO is expected to be present in the material as well; however, this is difficult to verify experimentally due to the overlapping peaks of MgO and Mg_3_Nd in XRD analysis.

Concerning the distribution of the secondary phases, they were found throughout the additively manufactured material. There is an accumulation of Mg-RE secondary phases along the melt pool boundaries which to some extent coincides with the grain boundaries. However, in cast material, precipitates are to a large extent found along the grain boundaries [12]. As has been observed in other studies on WE43 processed by PBF, the secondary phases are to a large extent found inside the grains. One reason for this could be the fast solidification of the material in the PBF-LB process, such that there is not enough time for the redistribution of the secondary phase to grain boundaries to occur.

Regarding the grain size and texture reported for Mg alloy WE43 processed by PBF-LB, the results vary between studies. Esmaily et al. [39] reported an average grain size of 17–36 µm. Moreover, the EBSD images in that study mainly show larger grains with a strong basal texture, similar to the results in this article for the samples produced with 120 J/mm^3^. Zumdick et al. [37], on the other hand, reported on a much smaller average grain size of 1.0–1.1 µm, while not mentioning anything about texture. Jauer et al. [65] reported that the observed microstructure mainly consisted of equiaxed grains having a grain size of around 1–5 µm and exhibiting only a weak texture. However, a few larger grains with a strong texture were observed. Bär et al. [36] investigated the grain size and texture in and around the final melt pool. They observed smaller equiaxed grains exhibiting a weak texture inside the melt pool, while small columnar grains with some texture were observed along the melt pool boundaries. Larger grains with a strong basal texture were observed in the previous layers that had been remelted and reheated as subsequent layers were added, indicating that grain growth took place. As opposed to many other additively manufactured materials, there was limited grain growth across the individual layers.

These results can be related to the resulting grain sizes and textures observed in the PBF-LB processed material in this study. As the beam spot size is 0.1 mm in the EOS M290, the hatch distance applied for the 120 J/mm^3^ sample was around one third of this (0.03 mm), compared to a 0.1 mm hatch distance for the 60 J/mm^3^ samples. This could mean that because several subsequent laser tracks overlapped for the 120 J/mm^3^ sample, the material was remelted more times in this sample, and thus fewer smaller grains are present. Nevertheless, the results show that, depending on the thermal conditions, two types of microstructures can be obtained. It should be noted that the use of ED as a measurement is not exact, and processing parameters having the same EDs could produce various densities. However, there is no other measurement of the amount of energy that is being introduced into the material. As the evaporation of magnesium is an important factor to consider and is closely related to the temperature of the melt, it is important to take the energy density into account.

Moreover, the alloying content of the different samples as obtained from the EDS mapping is not exact. However, the relative results are still relevant as input in order to study what happens in the thermodynamic model. Nevertheless, future studies should evaluate other methods in order to establish the exact changes in alloy content during PBF-LB.

Finally, in this study the gas flow was not optimized to avoid the removal of powder from the build plate during processing. However, the amount of powder being removed during processing was deemed insignificant due to the short time between the deposition of layers. Furthermore, laminar flow is needed to remove condensates formed during processing [65]. However, the effect of the gas flow and the deposition of the evaporation products on neighboring areas of the build plate should be further investigated. The amount of Mg evaporated in relation to the energy input should be more thoroughly investigated, as this can be expected to have an influence on the final alloy composition. A higher evaporation of Mg alloy would lead to a greater change in alloy composition and thus a higher number of intermetallic phases. However, no major differences could be established when comparing the intermetallic phases and their distribution in the two groups. In addition, no major differences in phase fractions were predicted using thermodynamic modelling. In summary, future work should include a larger study looking at the influence of individual process parameters and scanning strategies on the amount and distribution of secondary phases, grain size distribution, and texture. The resulting microstructures should be related to material properties, including corrosion behavior and mechanical strength. Moreover, with regard to the strong evaporation and large amount of condensation products formed in the process together with the strong affinity of magnesium for oxidation, conditions such as oxygen content in the build chamber, argon flow rate, and the positioning of the printed parts in relation to each other should be further investigated as well.

## 5. Conclusions

In summary, we have showed that it is possible to predict to a significant extent the phases formed in Mg alloy WE43 during PBF-LB processing by using the Scheil–Gulliver model. The difference in the optimized process parameters obtained with regard to minimizing porosity and avoiding overmelting did not have any major impact on the types of intermetallics formed nor on their distribution, with the exception of the amount of melt pool boundaries. However, the difference in process parameters did have an impact on grain size, grain size distribution and texture, and depending on the thermal conditions, two types of microstructures could be obtained. Here, the difference in grain size was mainly related to the hatch distance, as this affects the number of times the material was remelted. Finally, through this study the understanding of the influence of the PBF-LB process on a gas-atomized WE43 Mg alloy has been further enhanced, and the transferability of these results to future studies has been secured by the use of a standardized system.

## Figures and Tables

**Table 1:** Alloy composition as supplied by the manufacturer (NMD GmbH).

**Element (wt%)**	**Mg**	**Y**	**Nd**	**Zr**
	balance	3.9	3.0	0.49

**Table 2:** Printing parameters together with the resulting density of samples chosen for further evaluation (*n* = 5).

ED (J/mm^3^)	P (W)	V (mm/s)	H (mm)	Archimedes Density (%)
120	100	926	0.03	99.4 ± 0.05
60	200	1111	0.1	99.8 ± 0.2

**Table 3:** Chemical composition of the material processed with 120 J/mm^3^ and 60 J/mm^3^ as assessed by EDS and compared to the composition given for the powder.

	Element (wt%)			
	Mg	Y	Nd	Zr
Powder	balance	3.9	3.0	0.49
ED = 120 J/mm^3^	balance	5.4	3.8	0.6
ED = 60 J/mm^3^	balance	4.1	3.3	0.5

## Data Availability

The data presented in this study are available in this article.
